# Ethane-1,2-diaminium bis­(4-carb­oxy-2-propyl-1*H*-imidazole-5-carb­oxy­ate) monohydrate

**DOI:** 10.1107/S1600536812029406

**Published:** 2012-07-04

**Authors:** Yi-Mei Ying, Tong Zhang, Guang-Rui Yang, Ning Ma

**Affiliations:** a Institute of Environmental and Municipal Engineering, North China University of Water Conservancy and Electric Power, Zhengzhou 450011, People’s Republic of China; b Henan Museum, Zhengzhou 450001, People’s Republic of China

## Abstract

In the title hydrated molecular salt, C_2_H_10_N_2_^2+^·2C_8_H_9_N_2_O_4_^−^·H_2_O, an intra­molecular O—H⋯O hydrogen bond occurs in the anion, forming an S(7) ring. The –CO_2_ and –CO_2_H groups make dihedral angles of 3.2 (2) and 2.0 (3)°, respectively, with the five-membered ring. In the crystal, N—H⋯O, N—H⋯N and O—H⋯O hydrogen bonds lead to the formation of a three-dimensional supra­molecular architecture. The methyl group in the anion is disordered over two sets of sites in a 0.716 (9):0.284 (9) ratio. The ethylenediamine cation is generated by symmetry and the water molecule lies on a twofold axis.

## Related literature

For background to studies of supra­molecular structures of co-crystals containing organic acids and organic bases resulting from hydrogen bonding, see: Wang & Wei (2005 ▶).
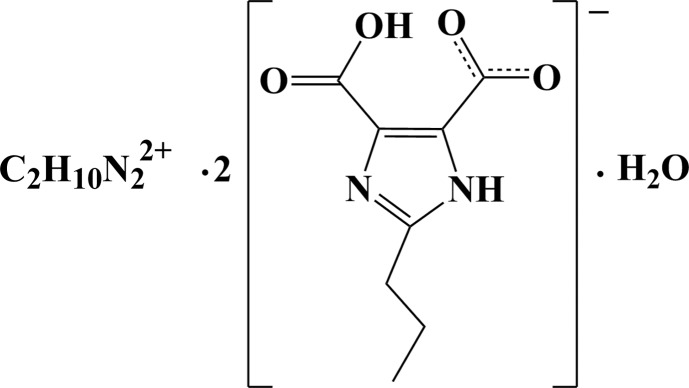


## Experimental

### 

#### Crystal data

C_2_H_10_N_2_^2+^·2C_8_H_9_N_2_O_4_^−^·H_2_O
*M* *_r_* = 474.48
Monoclinic, 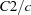

*a* = 15.234 (4) Å
*b* = 16.859 (4) Å
*c* = 9.699 (3) Å
β = 112.991 (5)°
*V* = 2293.1 (10) Å^3^
*Z* = 4
Mo *K*α radiation
μ = 0.11 mm^−1^
*T* = 296 K
0.36 × 0.28 × 0.16 mm

#### Data collection

Bruker SMART CCD area-detector diffractometer
Absorption correction: multi-scan (*SADABS*; Sheldrick, 2001 ▶) *T*_min_ = 0.961, *T*_max_ = 0.983
5444 measured reflections
2011 independent reflections
1500 reflections with *I* > 2σ(*I*)
*R*_int_ = 0.032

#### Refinement

*R*[*F* ^2^ > 2σ(*F* ^2^)] = 0.060
*wR*(*F* ^2^) = 0.182
*S* = 1.05
2011 reflections
169 parameters
34 restraints
H atoms treated by a mixture of independent and constrained refinement
Δρ_max_ = 0.30 e Å^−3^
Δρ_min_ = −0.45 e Å^−3^

### 

Data collection: *SMART* (Bruker, 2001 ▶); cell refinement: *SAINT-Plus* (Bruker, 2001 ▶); data reduction: *SAINT-Plus*; program(s) used to solve structure: *SHELXS97* (Sheldrick, 2008 ▶); program(s) used to refine structure: *SHELXL97* (Sheldrick, 2008 ▶); molecular graphics: *PLATON* (Spek, 2009 ▶); software used to prepare material for publication: *PLATON*.

## Supplementary Material

Crystal structure: contains datablock(s) global, I. DOI: 10.1107/S1600536812029406/gg2081sup1.cif

Structure factors: contains datablock(s) I. DOI: 10.1107/S1600536812029406/gg2081Isup2.hkl

Additional supplementary materials:  crystallographic information; 3D view; checkCIF report

## Figures and Tables

**Table 1:** Hydrogen-bond geometry (Å, °)

*D*—H⋯*A*	*D*—H	H⋯*A*	*D*⋯*A*	*D*—H⋯*A*
N2—H2*A*⋯O4^i^	0.86	1.92	2.759 (3)	166
N3—H3*A*⋯N1	0.89	2.03	2.921 (3)	176
N3—H3*A*⋯O1	0.89	2.54	2.964 (3)	110
N3—H3*B*⋯O1^ii^	0.89	1.94	2.792 (3)	160
N3—H3*C*⋯O1*W* ^iii^	0.89	2.08	2.917 (3)	157
O2—H2⋯O3	0.82	1.64	2.457 (3)	177
O1*W*—H1*W*⋯O3	0.85 (1)	1.96 (1)	2.795 (2)	169 (4)

Symmetry codes: (i) 
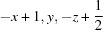
; (ii) 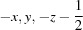; (iii) 
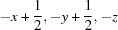
.

**Fig. 1.**
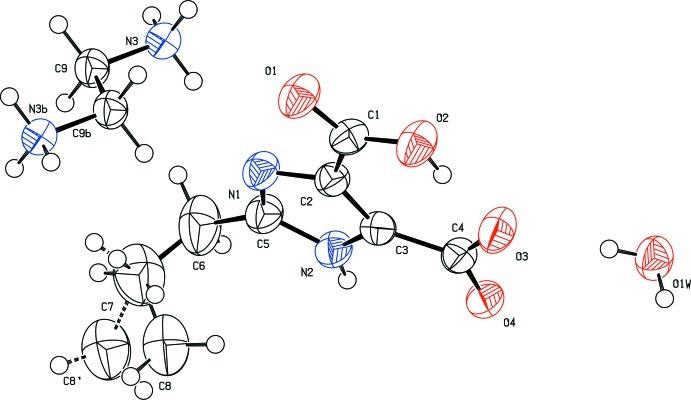
The molecular structure of (I), with displacement ellipsoids for the non-hydrogen atoms drawn at the 50% probability level.

**Fig. 2.**
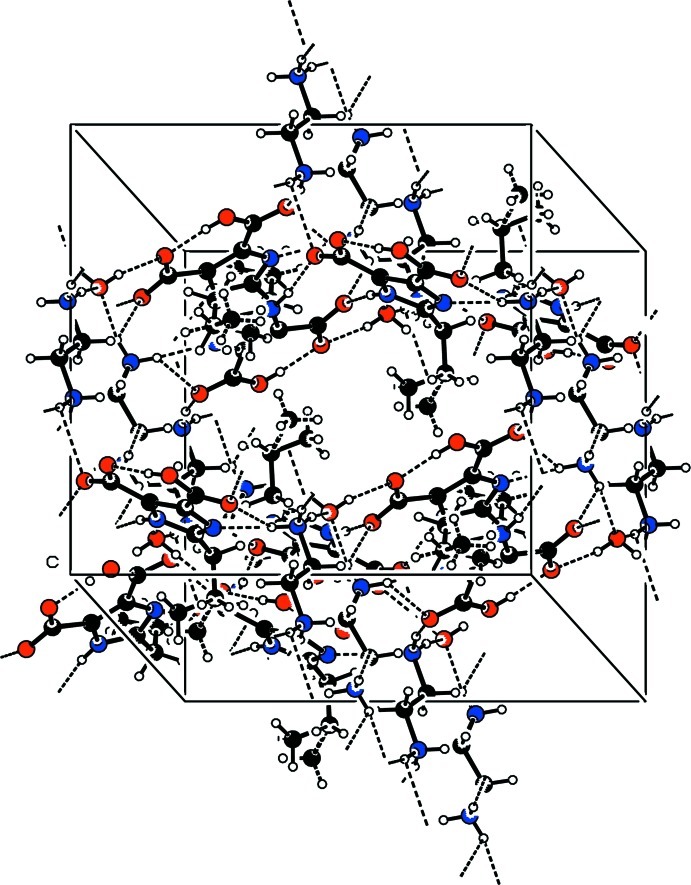
Three-dimensional structure of (I), with H-bonds indicated by dashed lines. Displacement ellipsoids for the non-hydrogen atoms are drawn at the 50% probability level.

## supplementary crystallographic information

### Comment

Currently, many groups are studying the supramolecular structures of co-crystals containing organic acids and organic bases resulting from hydrogen bonding (Wang & Wei, 2005).

The asymmetric unit of the title complex, (I), is composed of two 2-propyl-1*H*-imidazole-4-carboxylic acid-5-carboxyate anions, one diprotonated ethylenediaminium cation and one water molecule in general positions (Fig. 1). The C–O bond distances range from 1.221 (3)to 1.289 (3) Å, in which the C1–O1 [1.223 (3) Å], C4–O4 [1.221 (3) Å] and C4–O3 [1.266 (3) Å] are typical for C=O double bonds, whereas the C1–O2 bond length of 1.289 (3) Å indicates a C–O single bond. The elongation of the C4=O3 double bond is affected by the intra-molecular O2—H2···O3 hydrogen bonding interaction. Thus, the 5-carboxyl group of 2-propyl-1*H*-imidazole-4,5-dicarboxy acid is deprotonated, which must be balanced in charge terms by the presence of half of the diprotonated ethylenediamine. Furthermore, the acidic environment is propitious to the protonation of ethylenediamine.

In the crystal structure, intra-molecular hydrogen bonds are present, with O2–H2 acting as a hydrogen bond donor, and O3 atom as a hydrogen bond acceptor, thereby constructing S(7) rings. In addition, the diprotonated ethylenediaminium cations and 2-propyl-1*H*-imidazole-4-carboxylic acid-5-carboxyate anions together with water molecules are further linked into a three-dimensional supramolecular framework by multiple N—H···O, N—H···N and O—H···O hydrogen bonds (Fig. 2 and Table 1).

### Experimental

All reagents were commercially available and of analytical grade. The mixture of DyCl_3_.6H_2_O (0.189 g, 0.50 mmol), 2-propyl-1*H*-imidazole-4,5-dicarboxylic acid (0.197 g, 1.00 mmol), and ethylenediamine (1 ml) was dissolved in 50 ml H_2_O, and the mixture was stirred and heated to reflux at 80°C for two hours. The resulting solution was filtered, the filtrate was adjusted to pH = 7.5 using 4 *M* HCl solution, then was placed inside a programmable electric furnace at 130 °C for five days. After cooling the autoclave to room temperature, colorless block crystals of (I) were obtained.

### Refinement

H atoms were treated as riding, with C—H distances of 0.96 Å for methyl, 0.97 Å for methylene, N—H distances in the range of 0.96–0.89 Å and O—H distances of 0.82 Å for hydroxy group and 0.84 Å for water, and were refined as riding with *U*_iso_(*H*)=1.2*U*_eq_(C_methylene_, O2 and N) and *U*_iso_(H)=1.5*U*_eq_(O1W and (C_methyl_)).

### Crystal data

**Table:**

C_2_H_10_N_2_^2+^·2C_8_H_9_N_2_O_4_^−^·H_2_O	*F*(000) = 1008
*M**_r_* = 474.48	*D*_x_ = 1.374 Mg m^−^^3^
Monoclinic, *C*2/*c*	Mo *K*α radiation, λ = 0.71073 Å
Hall symbol: -C 2yc	Cell parameters from 356 reflections
*a* = 15.234 (4) Å	θ = 2.5–14.8°
*b* = 16.859 (4) Å	µ = 0.11 mm^−^^1^
*c* = 9.699 (3) Å	*T* = 296 K
β = 112.991 (5)°	Block, colorless
*V* = 2293.1 (10) Å^3^	0.36 × 0.28 × 0.16 mm
*Z* = 4	

### Data collection

**Table:**

Bruker SMART CCD area-detector diffractometer	2011 independent reflections
Radiation source: fine-focus sealed tube	1500 reflections with *I* > 2σ(*I*)
Graphite monochromator	*R*_int_ = 0.032
phi and ω scans	θ_max_ = 25.0°, θ_min_ = 1.9°
Absorption correction: multi-scan (*SADABS*; Sheldrick, 2001)	*h* = −18→17
*T*_min_ = 0.961, *T*_max_ = 0.983	*k* = −19→20
5444 measured reflections	*l* = −11→8

### Refinement

**Table:**

Refinement on *F*^2^	Primary atom site location: structure-invariant direct methods
Least-squares matrix: full	Secondary atom site location: difference Fourier map
*R*[*F*^2^ > 2σ(*F*^2^)] = 0.060	Hydrogen site location: inferred from neighbouring sites
*wR*(*F*^2^) = 0.182	H atoms treated by a mixture of independent and constrained refinement
*S* = 1.05	*w* = 1/[σ^2^(*F*_o_^2^) + (0.1098*P*)^2^ + 0.4118*P*] where *P* = (*F*_o_^2^ + 2*F*_c_^2^)/3
2011 reflections	(Δ/σ)_max_ < 0.001
169 parameters	Δρ_max_ = 0.30 e Å^−^^3^
34 restraints	Δρ_min_ = −0.45 e Å^−^^3^

### Special details

**Table:**

Geometry. All e.s.d.'s (except the e.s.d. in the dihedral angle between two l.s. planes) are estimated using the full covariance matrix. The cell e.s.d.'s are taken into account individually in the estimation of e.s.d.'s in distances, angles and torsion angles; correlations between e.s.d.'s in cell parameters are only used when they are defined by crystal symmetry. An approximate (isotropic) treatment of cell e.s.d.'s is used for estimating e.s.d.'s involving l.s. planes.
Refinement. Refinement of *F*^2^ against ALL reflections. The weighted *R*-factor *wR* and goodness of fit *S* are based on *F*^2^, conventional *R*-factors *R* are based on *F*, with *F* set to zero for negative *F*^2^. The threshold expression of *F*^2^ > σ(*F*^2^) is used only for calculating *R*-factors(gt) *etc*. and is not relevant to the choice of reflections for refinement. *R*-factors based on *F*^2^ are statistically about twice as large as those based on *F*, and *R*- factors based on ALL data will be even larger.

### Fractional atomic coordinates and isotropic or equivalent isotropic displacement parameters (Å^2^)

**Table:**

	*x*	*y*	*z*	*U*_iso_*/*U*_eq_	Occ. (<1)
N1	0.21377 (15)	0.37000 (13)	0.0929 (2)	0.0456 (6)	
N2	0.35960 (14)	0.32244 (13)	0.1911 (2)	0.0433 (6)	
H2A	0.4125	0.3057	0.2578	0.052*	
N3	0.00885 (15)	0.38982 (11)	0.0087 (2)	0.0402 (5)	
H3A	0.0712	0.3859	0.0313	0.048*	
H3B	−0.0229	0.3808	−0.0887	0.048*	
H3C	−0.0083	0.3542	0.0613	0.048*	
O1	0.10537 (13)	0.39974 (12)	−0.2035 (2)	0.0555 (6)	
O2	0.21678 (14)	0.35742 (13)	−0.2760 (2)	0.0566 (6)	
H2	0.2714	0.3404	−0.2372	0.068*	
O3	0.37886 (14)	0.30257 (13)	−0.1635 (2)	0.0578 (6)	
O4	0.48440 (12)	0.27422 (11)	0.0614 (2)	0.0509 (5)	
O1W	0.5000	0.20888 (16)	−0.2500	0.0544 (7)	
H1W	0.460 (2)	0.2394 (18)	−0.236 (4)	0.082*	
C1	0.18526 (18)	0.37268 (15)	−0.1732 (3)	0.0420 (6)	
C2	0.24741 (17)	0.35651 (13)	−0.0175 (3)	0.0381 (6)	
C3	0.33827 (17)	0.32705 (13)	0.0420 (3)	0.0383 (6)	
C4	0.40669 (18)	0.29940 (14)	−0.0224 (3)	0.0427 (6)	
C5	0.28372 (19)	0.34863 (17)	0.2174 (3)	0.0483 (7)	
C6	0.2826 (3)	0.3534 (2)	0.3692 (4)	0.0794 (11)	
H6A	0.2221	0.3324	0.3638	0.095*	
H6B	0.3321	0.3185	0.4343	0.095*	
C7	0.2958 (4)	0.4296 (3)	0.4395 (5)	0.1099 (14)	
H7A	0.2500	0.4671	0.3743	0.132*	0.716 (9)
H7B	0.2863	0.4261	0.5325	0.132*	0.716 (9)
H7C	0.2345	0.4451	0.4399	0.132*	0.284 (9)
H7D	0.3065	0.4642	0.3676	0.132*	0.284 (9)
C8	0.3826 (5)	0.4534 (3)	0.4659 (8)	0.095 (2)	0.716 (9)
H8A	0.3969	0.4436	0.3794	0.142*	0.716 (9)
H8B	0.4268	0.4248	0.5500	0.142*	0.716 (9)
H8C	0.3879	0.5091	0.4877	0.142*	0.716 (9)
C8'	0.3712 (10)	0.4585 (7)	0.5926 (14)	0.074 (4)	0.284 (9)
H8'A	0.4097	0.4994	0.5756	0.111*	0.284 (9)
H8'B	0.4110	0.4147	0.6435	0.111*	0.284 (9)
H8'C	0.3393	0.4791	0.6529	0.111*	0.284 (9)
C9	−0.0132 (2)	0.46962 (15)	0.0454 (3)	0.0439 (6)	
H9A	0.0219	0.4798	0.1513	0.053*	
H9B	−0.0807	0.4735	0.0245	0.053*	

### Atomic displacement parameters (Å^2^)

**Table:**

	*U*^11^	*U*^22^	*U*^33^	*U*^12^	*U*^13^	*U*^23^
N1	0.0374 (12)	0.0566 (13)	0.0414 (12)	0.0062 (10)	0.0139 (10)	0.0008 (10)
N2	0.0337 (11)	0.0519 (12)	0.0387 (12)	0.0060 (9)	0.0081 (9)	0.0002 (9)
N3	0.0384 (12)	0.0418 (12)	0.0415 (12)	0.0015 (9)	0.0168 (9)	0.0007 (8)
O1	0.0392 (11)	0.0788 (13)	0.0444 (11)	0.0144 (9)	0.0120 (8)	0.0084 (9)
O2	0.0443 (11)	0.0833 (14)	0.0414 (11)	0.0144 (10)	0.0160 (9)	0.0085 (9)
O3	0.0480 (11)	0.0825 (15)	0.0470 (12)	0.0123 (10)	0.0230 (9)	0.0021 (9)
O4	0.0334 (10)	0.0600 (11)	0.0520 (12)	0.0067 (8)	0.0088 (8)	−0.0088 (8)
O1W	0.0606 (19)	0.0557 (17)	0.0575 (17)	0.000	0.0347 (15)	0.000
C1	0.0373 (14)	0.0487 (14)	0.0387 (14)	−0.0001 (11)	0.0134 (11)	0.0057 (10)
C2	0.0347 (13)	0.0398 (13)	0.0390 (14)	0.0000 (10)	0.0135 (11)	0.0001 (10)
C3	0.0362 (13)	0.0375 (12)	0.0401 (13)	−0.0014 (10)	0.0137 (11)	−0.0004 (10)
C4	0.0388 (15)	0.0417 (13)	0.0482 (16)	−0.0007 (11)	0.0176 (12)	−0.0028 (11)
C5	0.0401 (14)	0.0608 (16)	0.0419 (15)	0.0049 (12)	0.0138 (12)	−0.0007 (12)
C6	0.070 (2)	0.121 (3)	0.0458 (18)	0.025 (2)	0.0207 (16)	−0.0054 (18)
C7	0.118 (3)	0.114 (3)	0.086 (3)	0.023 (3)	0.027 (3)	−0.003 (2)
C8	0.130 (4)	0.069 (3)	0.109 (5)	0.012 (3)	0.073 (4)	−0.005 (3)
C8'	0.108 (9)	0.052 (6)	0.061 (7)	0.002 (6)	0.030 (6)	−0.002 (5)
C9	0.0491 (15)	0.0442 (14)	0.0442 (14)	−0.0007 (11)	0.0245 (12)	−0.0034 (10)

### Geometric parameters (Å, º)

**Table:**

N1—C5	1.311 (3)	C6—C7	1.433 (6)
N1—C2	1.375 (3)	C6—H6A	0.9700
N2—C5	1.351 (3)	C6—H6B	0.9700
N2—C3	1.355 (3)	C7—C8	1.309 (7)
N2—H2A	0.8600	C7—C8'	1.558 (13)
N3—C9	1.464 (3)	C7—H7A	0.9700
N3—H3A	0.8900	C7—H7B	0.9700
N3—H3B	0.8900	C7—H7C	0.9699
N3—H3C	0.8900	C7—H7D	0.9698
O1—C1	1.223 (3)	C8—H7D	1.1934
O2—C1	1.289 (3)	C8—H8A	0.9600
O2—H2	0.8200	C8—H8B	0.9600
O3—C4	1.266 (3)	C8—H8C	0.9600
O4—C4	1.221 (3)	C8'—H8'A	0.9600
O1W—H1W	0.849 (10)	C8'—H8'B	0.9600
C1—C2	1.461 (4)	C8'—H8'C	0.9600
C2—C3	1.368 (3)	C9—C9^i^	1.505 (5)
C3—C4	1.484 (4)	C9—H9A	0.9700
C5—C6	1.481 (4)	C9—H9B	0.9700
			
C5—N1—C2	104.8 (2)	C6—C7—H7A	110.1
C5—N2—C3	108.4 (2)	C8'—C7—H7A	119.5
C5—N2—H2A	125.8	C8—C7—H7B	110.1
C3—N2—H2A	125.8	C6—C7—H7B	110.1
C9—N3—H3A	109.5	C8'—C7—H7B	57.0
C9—N3—H3B	109.5	H7A—C7—H7B	108.4
H3A—N3—H3B	109.5	C8—C7—H7C	144.9
C9—N3—H3C	109.5	C6—C7—H7C	106.7
H3A—N3—H3C	109.5	C8'—C7—H7C	105.3
H3B—N3—H3C	109.5	H7A—C7—H7C	51.7
C1—O2—H2	109.5	H7B—C7—H7C	61.3
O1—C1—O2	121.7 (2)	C8—C7—H7D	61.0
O1—C1—C2	120.0 (2)	C6—C7—H7D	103.2
O2—C1—C2	118.4 (2)	C8'—C7—H7D	103.3
C3—C2—N1	110.8 (2)	H7A—C7—H7D	54.6
C3—C2—C1	130.1 (2)	H7B—C7—H7D	146.5
N1—C2—C1	119.1 (2)	H7C—C7—H7D	105.9
N2—C3—C2	104.6 (2)	C7—C8—H7D	45.3
N2—C3—C4	121.1 (2)	C7—C8—H8A	109.5
C2—C3—C4	134.3 (2)	H7D—C8—H8A	79.0
O4—C4—O3	124.2 (2)	C7—C8—H8B	109.5
O4—C4—C3	119.2 (2)	H7D—C8—H8B	153.6
O3—C4—C3	116.6 (2)	C7—C8—H8C	109.5
N1—C5—N2	111.3 (2)	H7D—C8—H8C	89.9
N1—C5—C6	125.5 (3)	C7—C8'—H8'A	109.5
N2—C5—C6	123.2 (3)	C7—C8'—H8'B	109.5
C7—C6—C5	117.9 (4)	H8'A—C8'—H8'B	109.5
C7—C6—H6A	107.8	C7—C8'—H8'C	109.5
C5—C6—H6A	107.8	H8'A—C8'—H8'C	109.5
C7—C6—H6B	107.8	H8'B—C8'—H8'C	109.5
C5—C6—H6B	107.8	N3—C9—C9^i^	110.1 (2)
H6A—C6—H6B	107.2	N3—C9—H9A	109.6
C8—C7—C6	108.1 (5)	C9^i^—C9—H9A	109.6
C8—C7—C8'	53.5 (6)	N3—C9—H9B	109.6
C6—C7—C8'	130.3 (6)	C9^i^—C9—H9B	109.6
C8—C7—H7A	110.1	H9A—C9—H9B	108.2

Symmetry code: (i) −*x*, −*y*+1, −*z*.

### Hydrogen-bond geometry (Å, º)

**Table:**

*D*—H···*A*	*D*—H	H···*A*	*D*···*A*	*D*—H···*A*
N2—H2*A*···O4^ii^	0.86	1.92	2.759 (3)	166
N3—H3*A*···N1	0.89	2.03	2.921 (3)	176
N3—H3*A*···O1	0.89	2.54	2.964 (3)	110
N3—H3*B*···O1^iii^	0.89	1.94	2.792 (3)	160
N3—H3*C*···O1*W*^iv^	0.89	2.08	2.917 (3)	157
O2—H2···O3	0.82	1.64	2.457 (3)	177
O1*W*—H1*W*···O3	0.85 (1)	1.96 (1)	2.795 (2)	169 (4)

Symmetry codes: (ii) −*x*+1, *y*, −*z*+1/2; (iii) −*x*, *y*, −*z*−1/2; (iv) −*x*+1/2, −*y*+1/2, −*z*.
